# Transport Phenomena of Water in Molecular Fluidic Channels

**DOI:** 10.1038/srep33881

**Published:** 2016-09-21

**Authors:** Truong Quoc Vo, BoHung Kim

**Affiliations:** 1 School of Mechanical Engineering, University of Ulsan, Daehak-ro 93, Namgu, Ulsan 680-749, South Korea

## Abstract

In molecular-level fluidic transport, where the discrete characteristics of a molecular system are not negligible (in contrast to a continuum description), the response of the molecular water system might still be similar to the continuum description if the time and ensemble averages satisfy the ergodic hypothesis and the scale of the average is enough to recover the classical thermodynamic properties. However, even in such cases, the continuum description breaks down on the material interfaces. In short, molecular-level liquid flows exhibit substantially different physics from classical fluid transport theories because of (i) the interface/surface force field, (ii) thermal/velocity slip, (iii) the discreteness of fluid molecules at the interface and (iv) local viscosity. Therefore, in this study, we present the result of our investigations using molecular dynamics (MD) simulations with continuum-based energy equations and check the validity and limitations of the continuum hypothesis. Our study shows that when the continuum description is subjected to the proper treatment of the interface effects via modified boundary conditions, the so-called continuum-based modified-analytical solutions, they can adequately predict nanoscale fluid transport phenomena. The findings in this work have broad effects in overcoming current limitations in modeling/predicting the fluid behaviors of molecular fluidic devices.

The increasing importance of computational experiments has been recognized since the chemistry Nobel was awarded for the first time to three modelers (K. Karplus, M. Levitt, and A. Warshel) in 2013. Computer modelling has become an equal partner with experiments as joining together descriptions of molecules at close-up and zoomed-out scales^1^. During the past few decades, the transport phenomena of liquids in various molecular-level channels have been of great interest due to the emergence of mechanical and biochemical lab-on-the-chip systems, and nano-electromechanical system (NEMS) fabrication technologies^2^. More recently, well-controlled nanochannels in any desired shape and in a variety of geometric dimensions could be produced using the ‘crack-photolithography’ technique, which is very promising for nanofluidic applications^3^,^4^.

Although the discrete characteristics of molecular systems are not negligible (in contrast to the continuum description), the response of the molecular water system might be similar to the continuum model if the ergodic hypothesis develops and the scale of average is large enough to recover the classical thermodynamic properties (usually several tens of molecular diameter length scale). The ergodic hypothesis indicates that the time average value of an observable is equivalent to an ensemble average, that is, an average at one time over a large number of systems. Even in such cases, the continuum description still breaks down on the material interfaces. It has been well established that the surface effects become dominant in nano-conduit geometries because their dimensions are comparable to the force penetration distance induced by the wall molecules^5^,^6^,^7^,^8^,^9^,^10^,^11^,^12^,^13^,^14^,^15^,^16^. The size effects therefore render the no-slip boundary condition inapplicable at the micron and sub-micron scales. Consequently, the introduction of velocity slip length (*L*_s_) and Kapitza length^17^ (*L*_K_) lead to the breakdown of continuum models^2^,^18^,^19^. On the other hand, experimental evidence has shown that the dynamic structuring of liquid molecules in the proximity of a solid surface advances as a series of distinct molecular layers^20^,^21^,^22^,^23^,^24^,^25^. The formation of liquid layers induces a non-uniform variation of viscosity inside the channel and thus profoundly influences the interfacial transport, as demonstrated by both experiments^26^,^27^,^28^ and numerical methods^29^,^30^,^31^,^32^,^33^,^34^,^35^,^36^. Thus far, the substantially different physics between fluid flows at molecular-levels and in continuum models can be interpreted using four factors: (i) interface/surface force field, (ii) thermal/velocity slip, (iii) discreteness of fluid molecules at the interface and (iv) local viscosity. An overview of the density, velocity, viscosity, and temperature profiles of liquid flows from continuum-level assumptions and with molecular-surface effects is shown in Fig. 1a.

For the reasons just given, proper evaluation and control of interfacial transport at the molecular-level has become a challenge and crucial demand for scientists. Therefore, in this study, we present the result of our investigations using molecular dynamics (MD) simulation method with continuum-based energy equations and check the validity of the continuum hypothesis. We analytically solve the problem of nanoscale transport phenomena with the help of the continuum approach and boundary treatment. Importantly, we clarify the contribution of interface effects, especially the role of near-wall viscosity, which has not been thoroughly studied in the context of molecular-level fluid transport. Despite the well-studied interfacial phenomena between liquid water and graphene or carbon nanotubes (CNTs)^33^,^34^,^35^,^36^, the effects of graphene-coated copper surfaces on the molecular behavior of water have yet to be fully elucidated. The unique structures and properties of graphene have attracted tremendous interest for a wide range of application in nano-devices. Recent developments in nanoscience and nanotechnology have made it possible to deposit mono-/few-layer graphene on bare substrates^37^,^38^,^39^. Although graphene is one atom thick, it can have the energy transmission from the underlying solid substrate to the thin film liquid changed^40^,^41^,^42^. Therefore, the introduction of graphene leads to a new class of surfaces, the so-called nanocomposite walls, at which the wall-fluid interaction strength is tuned. This coating method overcomes the limitations of the recent reports^5^,^6^,^7^,^8^,^9^,^10^,^11^,^14^,^30^,^31^ in which the interface physics have been investigated by mainly changing the wall-fluid interaction strength. Instead of such simple variations, the interaction parameters between non-identical pairs need to be reparameterized in such a way that the wetting behavior can be recovered. In the present research, we study the coupled momentum and heat transfer in molecular-level channel shear-driven water flow at Cu (111) surfaces with and without a single graphene layer. We aim to not only study the viscous heating and resulting temperature profiles of the confined fluids, but also compare and contrast the MD results with the continuum prediction. We dedicate particular attention to local variations in fluid density, velocity, viscosity, and temperature, which play key roles in continuum modeling of viscous heat dissipation based on energy conservation. We begin by modeling water droplets to identify the wettability of bare and composite solid surfaces. Next, we consider 50-m/s shear-driven 7 nm-high water flow channels to measure the flow properties. The results obtained from the driven flow simulations could be utilized to the general flow problems because the applied shear rate is in the linear regime^5^. More details on the simulation technique can be seen in the Simulation Methods section.

## Results and Discussion

### Control of surface wettability and energy using coating method

**Figure 1:**
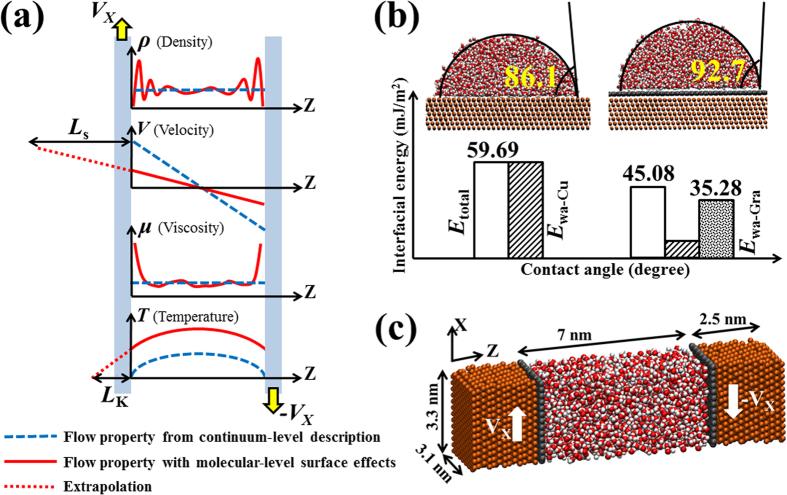
Schematic of boundary conditions and molecular dynamics (MD) simulation setup. (**a**) Overview of fluid density, velocity, viscosity, and temperature profiles from continuum-level description and with molecular-surface effects. The slip length (*L*_s_) and Kapitza length (*L*_K_) are defined as the distance between the wall-fluid interface and the point where the velocity and temperature vanish, respectively. (**b**) Cu and graphene-coated Cu surface wettability are determined by measuring the contact angles from water droplet simulations. The total solid-liquid interfacial energies (*E*_total_) with the contributions of water-Cu (*E*_Wa-Cu_) and water-graphene (*E*_Wa-Gra_) for corresponding cases are calculated using equation (1). (**c**) Couette flows of water in graphene-coated Cu nanochannel 7-nm-high with a shearing velocity of 50 m/s.

**Figure 2:**
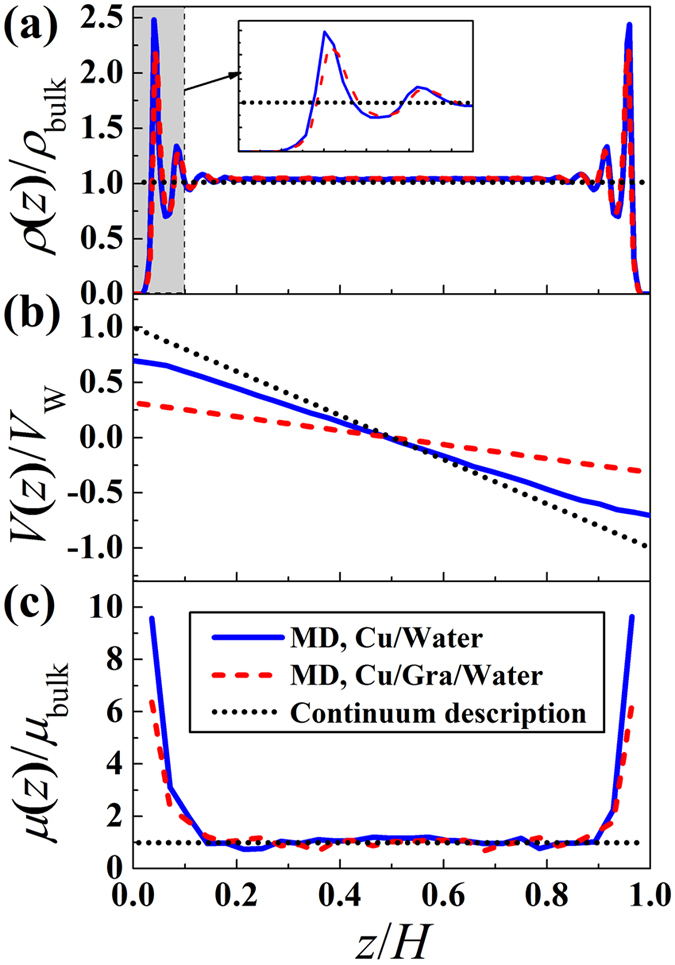
How molecular-level flows exhibit substantially different physics from continuum descriptions. (**a**) Water density distribution adjacent to solid surfaces. (**b**) Couette flow velocity profiles. (**c**) Viscosity variation across the channel. The solid and dashed lines are the simulation-measured flow properties inside the Cu and graphene-coated Cu channels, respectively. The dotted line represents the continuum description.

The wettability is determined by measuring the contact angles between water droplets and solid substrates, as shown in Fig. 1b. Clearly, the graphene coating enhanced the surface hydrophobicity. The obtained wetting angles are consistent with recent experimental studies^37^,^38^,^39^ and thus validate the intermolecular interactions we used in this study. The variations in surface wettability suggest that depositing the graphene sheet modified the Cu-water binding energy. To further explain the wetting changes, we calculated the total energy per unit area (*E*_total_) as a function of the separation between the water and the surface (*h*) and the thickness of the graphene film (*d*, including the interlayer spacing between the graphene and Cu) using the following equation^43^,^44^,^45^:


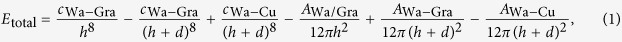


where the short-range repulsive interactions, *c*, and the Hamaker constant, *A*, are taken from ref. 37 corresponding to macro-scale behaviors as *c*_Wa-Gra_ = 0.98 × 10^−80^ Jm^6^, *c*_Wa-Cu_ = 2.52 × 10^−80^ Jm^6^, *A*_Wa-Gra_ = 9.08 × 10^−20^ J, *A*_Wa-Cu_ = 12.2 × 10^−20^ J. Meanwhile, we obtained *h*, defined as the point at which the water density exhibits half of the bulk value (0.5 g/cm^3^), from our simulation results for liquid density distributions (see Fig. 2b). We found a separation of 2.26 and 2.35 Å for the water-Cu and water-graphene interfaces, respectively. Figure 1b additionally represents the dependence of the graphene coating on *E*_total_ with the percentage of the contribution of each pair: water-Cu (*E*_Wa-Cu_) and water-graphene (*E*_Wa-Gra_). The adsorption energy values numerically calculated in this study showed good agreement with those from experiments^46^. Thus far, the wetting variations of solid surfaces can be interpreted from the aspect of surface energy. The reduction of *E*_total_ following the graphene deposition is mainly attributed to the less-dominant effects of Cu on water. Indeed, the influence of Cu-water interactions was significantly reduced when adding mono-layer graphene.

### Density, velocity, and viscosity distributions across the channel

To measure the thermal/velocity slip and local viscosity, we performed MD simulations of shear-driven water flows, as shown in Fig. 1c. However, an important first step in predicting the mechanism of water flows is clarifying the molecular structure of water molecules inside the channels. The strong density oscillations of the fluid molecules in the vicinity of solid surfaces is a universal phenomenon, and it has been observed in both the graphene-coated and non-coated Cu surfaces, as shown in Fig. 2a. Those oscillations decay and converge to the bulk-like value (1 g/cm^3^) further on the interface, at a distance of about 0.8 nm, leading to various peaks and valleys through the combination of attractive and repulsive forces between particles. However, at a short distance from the solid surface, fluid density was essentially zero due to the strong repulsion between particles. The discreteness of water molecules is profoundly affected by the neighboring wall molecules. Although the layered arrangements of liquid are not prominent in most macroscopic problems, they cannot be neglected when studying nanoscale thermal fluidic transport. The fluid dynamics at such small scales is strongly influenced by variations on the interface and the flow boundary layer because the liquid molecules can be captured to the wall and behave as an extended wall layer in this region, causing non-homogeneous distribution of flow properties. However, this inhomogeneity of the fluid has not been accounted for in classical fluid transport theories. On the other hand, the first peak density of liquid water on the graphene sheet exhibited a smaller value than that same peak on the Cu surface. This observation supports the conclusion that the presence of graphene has modified the wall-fluid binding energy, which in turn influences the adsorbed layer.

Figure 2b shows the steady-state velocity fields induced by translating the walls. We found that the profiles are strongly affected by the wall properties, even under the same shear rate. In the cases studied here, interfacial slip developed. Cieplak *et al*. have suggested that the slip length of the Couette flow is independent of the type of flow or channel height but that it is a strong function of the wall type^10^. Therefore, the variations in velocity profiles are caused by the fact that the amount of momentum transfer at the interface decreases with the decreasing solid-liquid intermolecular potential, which is caused by the graphene coating. For example, the interface between water and the bare Cu surface, which exhibits a stronger solid-liquid binding energy, shows almost similar velocities for fluid molecules and wall molecules, which implies that the fluid molecules were dynamically structured and attached to the wall and partly traveled with the wall molecules.

When fluid is confined in nanochannels, the fluid viscosity near the interface can differ significantly from the bulk value because of the surface force and fluid layering effects. As shown in Fig. 2c, the results demonstrated the apparent enhanced viscosity. The local dynamic viscosity of liquid water is defined as the ratio between the local shear stress and the applied shear rate. Details on computations of the shear components of the stress tensor for a certain atom can be seen in the Simulation Methods section. To the mechanical equilibrium aspects, the shear stress remains constant along the confined channel. The interpretation is that the molecules collide as hard spheres and transfer momentum only through direct collisions. For such cases, thermostat is only applied to the fluid to maintain the system at thermal equilibrium, and therefore, heat transfer caused by viscous heating is completely ignored. Such MD models have either physical or computational limitations in coupled momentum and heat transfer simulations. To enable the viscous heating induced by the shearing motion, the solid walls were used as the heat reservoirs in the present study. As a result, energy imparted on the fluid is dissipated by itself, while the constant wall temperature applied on the surface allows heat loss through the wall. Under those conditions, the shear stress distributions obtained from our MD simulations exhibited a tendency similar to the density distributions. Specifically, the stress fluctuated in the vicinity of the surface. Those fluctuations gradually became less notable and converged to the bulk value in the mid-flows. We attribute this result to the fact that the molecules additionally interact through a certain range force-field, which extends through a few molecular diameters, leading to stress distributions around each molecule. This phenomenon is particularly visible when the wall molecules have much stronger intermolecular force fields.

### Continuum-based modified-analytical solution

To apply the continuum analysis for solving the temperature distribution induced by viscous heat dissipation based on energy conservation, we used the energy equation from the conservation law for 1-D shear-driven Newtonian fluid flow:


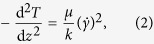


where *T* is the temperature, *μ* is the viscosity, *k* is the thermal conductivity and 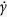 is the shear rate. The boundary conditions for equation (2) are 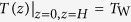, where *H* is the channel height, and *T*_W_ is the wall temperature. Hence, the resulting temperature distribution inside a channel can be estimated as


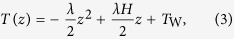


where 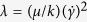. Equation (3) appropriately predicts the temperature profile on continuum levels. However, in nanoscale conduits, the surface effects must be taken into account for the boundary conditions. As shown previously, the viscosity in a molecular-level channel exhibits a non-homogenous distribution. Beyond several atomic diameters from the solid surface, water molecules do not interact directly with the solid, exhibiting bulk-like hydrogen-bonding characteristics. Near the solid surface, however, interactions with the wall atoms affect the dynamics of water molecules causing the formation of liquid layers. Consequently, transport properties in this region deviate from those of bulk water. Therefore, we considered the size-dependent viscosity—the effective viscosity (*μ*_eff_)—as follows:


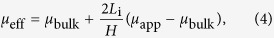


where *η*_bulk_ is the bulk viscosity of liquid water (*η*_bulk_ = 0.71 Pa.s × 10^−3^), and *L*_i_ is the solid-liquid interface thickness corresponding to 0.8 nm for this work. Similar thicknesses for water-graphene and water-copper interfaces have been found recently^23^,^35^,^36^,^41^. We calculated the apparent viscosity, *μ*_app_, as the average of local viscosity within the interface region. According to equation (4), *μ*_eff_ increases dramatically at molecular-level channel height, whereas *μ*_eff_ reduces to *μ*_bulk_ for 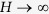. It has been reported that the effective viscosity is significant in nanoscale conduits, and therefore, once the effective viscosity is explicitly defined, the continuum theories using the effective viscosity can predict the macroscopic observables correctly^2^.

On the solid-liquid interfaces, the nanoscale heat transfer is mainly affected by the interfacial thermal resistance, resulting in a temperature jump Δ*T*. As a result, *L*_K_ can be predicted as 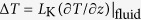, where 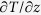 is the thermal gradient on the fluid side. Therefore, the boundary conditions for equation (3) with considerations of thermal slip are modified following 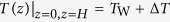, and thus, we resolve equation (3), which yields:


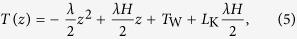


where *L*_K_ plays the same role in heat transfer as *L*_s_ in momentum transfer. *L*_s_ is the exact analog of *L*_K_, and they are interdependent quantities^14^,^19^. Thus, the introduction of *L*_K_ in continuum models is equivalent to that of *L*_s_.

**Figure 3:**
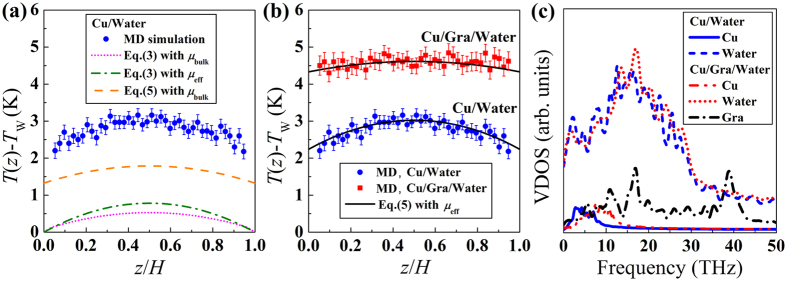
Solution for predicting temperature distribution across the molecular-level channel. (**a**) Temperature distribution of water across the Cu channel significantly deviates from the continuum energy equation (dotted line), one subjected to the effective viscosity (dash-dotted line), and one considering the thermal/slip boundary condition (dashed line). (**b**) The continuum-based modified-analytical solution, which is subjected the proper treatment of the interface effects, shows good qualitative agreement with the temperature measured from MD simulations. (**c**) The vibrational density of states (VDOS) of Cu, water, and graphene. Cu VDOS is collected from the outermost layer. For the liquid, the VDOS is calculated for molecules within 0.8 nm of the wall-fluid boundary. The average temperature obtained from five dependent simulations for each case is plotted with error bars showing the standard deviation in **a** and **b**.

We next start to predict the temperature profiles. We applied the continuum descriptions subjected to the proper treatment of interface effects into the modified boundary conditions to solve the resulting temperature distributions. Here, we assume the thermal/velocity slip and effective viscosity to be the main consequences of the size effects. To verify the contribution of each factor to the solutions, we sequentially considered and subjected each one to the continuum and modified-continuum predictions and compared the results with the simulation-measured temperature profiles, as shown in Fig. 3a. The dotted line is obtained by solving equation (3), which assumes the no-slip boundary condition and constant viscosity. It is clear that the classical theory completely failed to describe the thermal dynamics of fluid flows past a surface. Meanwhile, the dash-dotted line is obtained by solving the same equation using the effective viscosity, which showed better prediction at the mid-flow. Whereas, the data labeled as “Eq. (5) with *μ*_bulk_”, which considers the interfacial thermal/velocity slip but assumes constant viscosity, more approached to the simulated temperature profile at both the interface and centre of the channel. Nonetheless, we still found significant deviations. The underestimation of the predicted temperature profiles in Fig. 3a stems from the inaccurate assumptions of the no-slip boundary condition and the constant shear viscoisty across the channel. Therefore, we can account for the enhanced viscosity effect by solving equation (5) using the calculated effective viscosity as shown in Fig. 3b. The results indicated that the predicted temperature profile shows good qualitative agreement with the temperature measured from MD simulations. Therefore, the effects that the effective viscosity and the slip boundary condition have on temperature profiles were confirmed. Figure 3b additionally shows that the presence of graphene modified the solid-liquid potential energies, leading to variations in the amount of energy and momentum transport and, consequently, to the temperature distributions. A broader temperature profile and larger temperature jump were observed in the Cu/graphene/water system because of the reduced surface energy.

On the other hand, we calculated the vibrational density of states (VDOS), i.e., number of vibrational modes per unit volume and frequency, by performing a Fourier transform of the velocity autocorrelation function (VACF) to provide a better understanding of interfacial thermal transport. We calculated VDOS for the outermost Cu layer, graphene layer, and water molecules adjacent to the solid surface (Fig. 3c). More details on computations of VACF and VDOS can be found in the Simulation Methods section. We observed an insignificant spectrum change for water at different interfaces. However, the VDOS in the outermost Cu layer shifted toward to the high frequency range in the presence of graphene. In addition, the overlap between the VDOS of interfacial water and the solid surfaces was enhanced when graphene was deposited. Typically, a good overlap between the spectra implies strong vibrational coupling and, consequently, low temperature jump. However, referring to the results in Figs 2 and 3, we infer that the variations in *L*_K_ (or *L*_s_) do not develop from the change in VDOS. In other words, the momentum transfer at the solid-liquid interface is independent of VDOS; the wall-fluid binding energy and adsorbed liquid layer are the crucial factors. This finding differ highly from the case of solid−solid interface, where the overlap between the VDOS regulates the heat transfer^47^. A similar observation has been reported^48^.

## Conclusions

In summary, our theoretical and numerical findings provide clear evidence that the classical continuum theory cannot predict the transport phenomena at molecular solid-liquid interfaces, but the dynamic behaviors of the bulk fluid are very similar to the classical theory. These observations follow from the fact that the nanoscale flows exhibit significantly different physics from continuum models because of (i) the interface/surface force field, (ii) thermal/velocity slip, (iii) the discreteness of fluid molecules at the interface and, (iv) local viscosity. When the continuum description is subjected to the proper treatment of the interface effects via modified boundary conditions, the so-called continuum-based modified-analytical solutions, they can adequately predict nanoscale transport phenomena via the continuum hypothesis. Those surface-dominated and non-continuum effects diminish in length and time scales incomparably larger than the molecular free path and molecular relaxation time^2^. The results in this work can help overcome current limitations in modeling/predicting the fluid behaviors of nanofluidic devices.

## Simulation Methods

We employed MD simulation to study the transport phenomena of water in molecular fluidic channels. We deposited a single graphene layer on the Cu (111) surface with a Cu-graphene interlayer spacing of 2.9 Å. We used the same lattice constant of 2.552 Å to model both the Cu (111) and graphene. We used the many-body potential EAM^49^ to model the Cu-Cu interactions. Meanwhile, we simulated the interactions between C atoms in the graphene layers by the AIREBO potential^50^. The Cu substrate in droplet simulations was formed by seven layers of Cu (111). On the other hand, the Cu walls for generating the shear-driven flows were built using 13 layers of Cu (111). We used the SPC/E water model composed of Lennard-Jones (LJ) and Coulombic potential terms for the liquid^51^. A particle-particle particle-mesh (PPPM) was used to calculate the long-range electrostatic force^52^. The harmonic O-H bond length and the H-O-H angle were kept rigid using the SHAKE algorithm^53^. We used the truncated LJ (12-6) potential to model the vdW interactions as follows


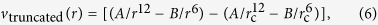


where 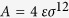, 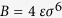, *ε* is the depth of the potential well, *σ* is the molecular distance at which the interatomic potential is zero, *r* is the intermolecular distance, and *r*_c_ is the cut-off. The intermolecular interactions of Cu-O, C-O, and Cu-C were additionally represented by the LJ potential. Specifically, *ε*_Cu-O_ = 0.0114563 eV, *σ*_Cu-O_ = 2.75185 Å, *ε*_C-O_ = 0.00412 eV, *σ*_C-O_ = 3.19 Å, *ε*_Cu-c_ = 0.02578 eV and *σ*_Cu-C_ = 3.0825 Å. LJ potential is one of the most widely used potentials for nonpolar atoms or molecules. Since it can represent the essential physics as combining realistic description of the intermolecular interaction and computational simplicity^2^,^52^. For both the water droplet and shear-driven water flow simulations, we applied the periodic conditions in the *x* and *y* directions. The outermost layers were fixed to their original positions to maintain a system of constant volume. We started from the Maxwell-Boltzmann velocity distributions for all molecules at 300 K. The systems were maintained at 300 K using Nose-Hoover thermostats with NVT ensemble for 2.0 ns. Then, we subjected the Langevin thermostats to the outermost second and third Cu layers. Simultaneously, the NVE ensemble was applied. In this state, we performed simulations for 6 ns, with the first 2 ns allowed for the systems to reach the steady state, and the rest for data collection. The simulation time step was set at 1.0 femtosecond (fs). We carried out all MD simulations using the LAMMPS package^54^.

Computations of the atomistic stress tensor included the two additive components: kinetic and virial terms. The kinetic term calculates the linear momentum resulting from particle velocities, while the virial term considers the internal contribution from short-range van der Waals forces, long-range Coulombic interactions, and the internal constraint forces of the bonds and angles of water molecules^55^,^56^. For instance, the stress tensor for atom *i* are given by the following formula^57^


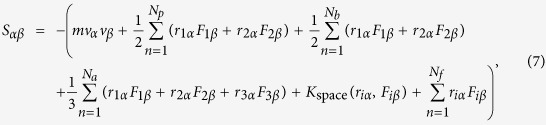


where the first term on the right-hand side is the kinetic component; *m* is the atomic mass of particle *i*; and *v*_*α*_ and *v*_*β*_ are the velocity components of particle *i* in the *α* and *β* directions, respectively. The second to fifth terms are virial components consisting of: (1) the second term is a pairwise energy contribution where *n* loops over the *N*_*p*_ neighbors of atom *i*, *r*_1_ and *r*_2_ are the positions, and *F*_1_ and *F*_2_ are the forces of the two atoms in the pairwise interaction; (2) the third and the fourth terms are the bond and angle contributions for the *N*_*b*_ bonds, and the *N*_*a*_ angle of which atom *i* is part of, respectively; (3) the *K*_space_ term is the contribution from the long-range Coulombic interactions for PPPM solver; and (4) the fifth term is the SHAKE internal constraint force applied to particle *i* via the *N*_*f*_ fixes. The per-atom array values listed above are the products of the stress and volume units. Therefore, the local shear stress in each slab bin (*S*_XZ_) positioned parallel to the walls was achieved by dividing the average of the total per-atom stress tensor by the particular volume of the slab bin.

MD can be utilized to compute the VDOS of atoms using the Fourier transform of its VACF as^58^


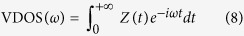


where the VACF is defined as 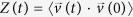.

## Additional Information

**How to cite this article**: Vo, T. Q. and Kim, B. Transport Phenomena of Water in Molecular Fluidic Channels. *Sci. Rep.* **6**, 33881; doi: 10.1038/srep33881 (2016).
